# Association between consumption of nonessential energy-dense food and body mass index among Mexican school-aged children: A prospective cohort study

**DOI:** 10.21203/rs.3.rs-2833950/v1

**Published:** 2023-04-26

**Authors:** Tonatiuh Barrientos-Gutiérrez, Daniel Illescas-Zárte, Carolina Batis, Gitanjali Singh, Dariush Mozaffarian, Ivonne Ramirez, Albino Barraza-Villarreal, Isabelle Romieu

**Affiliations:** National Institute of Public Health; National Institute of Medical Sciences and Nutrition Salvador Zubirán; Tufts University; Tufts University; Tufts University; National Institute of Public Health; National Institute of Public Health; IARC, France

## Abstract

**BACKGROUND/OBJECTIVES:**

Obesity prevalence in Mexican children has increased rapidly and is among the highest in the world. We aimed to estimate the longitudinal association between nonessential energy-dense food (NEDF) consumption and body mass index (BMI) in school-aged children 5 to 11 years, using a cohort study with 6 years of follow-up.

**SUBJECTS/METHODS:**

We studied the offspring of women in the Prenatal omega-3 fatty acid supplementation, child growth, and development (POSGRAD) cohort study. NEDF were classified into four main groups: chips and popcorn, sweet bakery products, non-cereal based sweets, and ready-to-eat cereals. We fitted fixed effects models to assess the association between change in 418.6 kJ (100 kcal) of NEDF consumption and changes in BMI.

**RESULTS:**

Between 5 and 11 years, children increased their consumption of NEDF by 225 kJ/day (53.9 kcal/day). In fully adjusted models, we found that change in total NEDF was not associated with change in children’s BMI (0.033 kg/m^2^, [p=0.246]). However, BMI increased 0.078 kg/m^2^ for every 418.6 kJ/day (100 kcal/day) of sweet bakery products (p=0.035) in fully adjusted models. For chips and popcorn, BMI increased 0.208 kg/m^2^ (p=0.035), yet, the association was attenuated after adjustment (p=0.303).

**CONCLUSIONS:**

Changes in total NEDF consumption were not associated with changes in BMI in children. However, increases in the consumption of sweet bakery products were associated with BMI gain. NEDF are widely recognized as providing poor nutrition yet, their impact in Mexican children BMI seems to be heterogeneous.

## Introduction

In the last four decades, the prevalence of obesity in children has increased in developing countries from 8.3–13.2% [1]. In Mexico, the prevalence of obesity in school-aged children has increased rapidly from 9.0% in 1999 to 18.6% in 2021 [1–4]. Childhood obesity has been linked to lower quality of life, higher risk of non-communicable diseases in adulthood, such as hypertension, dyslipidemia, type-2 diabetes, and premature death [5–8]. The hypothesis is that a low-quality diet, rich in nonessential energy-dense food (NEDF), often termed “junk food”, “discretionary food” or “processed food”, is a key factor for weight gain and obesity [9–11]. NEDF could be a critical risk factor for obesity in children and adolescents in Mexico as they displace healthy foods [12], are high in sugar, refined grains, unhealthy fats [10, 13], and have a high glycemic index [12].

NEDF consumption, defined in previous studies as energy-dense food without a cut-off point or > 13% of total energy from added sugars and/or saturated fat is high in Mexico, particularly among school-aged children living in big cities [10, 14, 15], who on average consume 21% of their total daily energy requirement from NEDF, with 50% of this consumed at school [14, 15]. School-aged children are particularly vulnerable to NEDF consumption due to targeted marketing and advertisement to young children as well as misleading nutritional information [16, 17]. Increased access to NEDF in this age group was large due to high availability in elementary schools and school-aged children have not fully developed their cognitive capacity of resistance towards these foods, and are less able to avoid or reduce their consumption [18].

Although NEDF might play a critical role as a risk factor for childhood obesity, due to their low diet quality and high intake among children, evidence of the effect of NEDF is limited. One of the main limitations is the lack of a consistent definition or classification of NEDF. Moreover, longitudinal studies of the effect of several NEDF classifications to weight gain in children are scarce and with mixed results [19–23]. Some of the limitations identified in those studies may be small samples sizes, short follow-ups, and limited information on changes in NEDF consumption over time, which could explain the lack of consistent findings. Further evidence on the potential link between NEDF consumption and weight gain in children is needed, to understand the impact of these food group in child wellbeing and health. Our aim was to estimate the longitudinal association of the change in NEDF consumption over time and changes in body mass index of school-aged children 5 to 11 years of age, using a cohort study with 6 years of follow-up.

## Methods

### Study design and population

We studied the offspring of women in the ongoing POSGRAD (Prenatal omega-3 fatty acid supplementation and child growth and development) study a double-blind, randomized, controlled trial in which women were supplemented with DHA or placebo from mid-pregnancy to parturition. The original study included 978 live births between June 2005 and June 2007 to 973 women who remained in the study as previously described [24]. Mother-child pairs have been followed prospectively. All children in the cohort had access to the Mexican Social Security Institute in Cuernavaca, Mexico, which provides services to formal employees and their families.

### Study variables

#### Outcome variables

Weight and height were measured twice by trained personnel using standardized procedures [25]; the average of both measurements was used. Weight with light clothes or a hospital gown was measured to the nearest of 100 g using a digital step-up scale (SECA 803). Height was measured without shoes, hat, hairclips, headbands or other items that could obstruct the procedure using a portable stadiometer (SECA 213). Then we obtained body mass index (weight_(kg)_/height_(mts)_^2^, BMI) and calculated z-scores based on the WHO growth reference [26, 27].

##### Exposure variables

Dietary intake was evaluated at the three waves. At baseline a 24-hour recall (24HR) questionnaire was applied by a trained interviewer to the person in the household who prepared the meals (frequently the mother); for waves 2 and 3 the same 24HR standardized method were applied but through an automated software previously used in a National Survey [28]. This is a method which capture more accurate information of the interviewees through 5 iterative steps that complement each other for memory improvement in food intake and thus, reducing under-reporting [29]. During the interview, children participated in the report of their diet, complementing, and validating their mothers’ report and in some cases adding missing food, correcting the size of a portion or removing the food reported by the adult. 24HR were performed from Sunday to Friday. To increase the accuracy of portions size, we used standardized food replicas, images of products, spoons, and cups of different sizes. Dietary information was collected as follows: 1) individual foods, 2) custom recipes (recipe reported and described in detail by the participant), and 3) standard recipes (set of ingredients in a documented and standard recipe when unknown to the subject). For our analysis all the recipes were disaggregated into their ingredients (with exception of beverages) to facilitate identifying all NEDF in recipes, (e.g. chips, puffed wheat snacks, candies, chocolate, sweets, others).

To address outliers in food items, we identify those when the reported amount was > 4 SD from the mean for the same food and age group to minimize their influence in total diet and analysis. We identify less than 0.1% as outliers and were truncated at the highest value (median + four SD) to minimize their influence in total diet and analysis. We did not identify implausible reporters by using the ratio for total energy intake to estimated energy requirement out of the interval between − 3 and + 3 SD in each wave. After the first stage of data cleaning and processing, energy, nutrients and added sugar from food were obtained with the Mexican Food Database in its 18.1.1version that include 1978 different foods including standardized recipes, and labeling information from some processed products [30].

Total NEDF was classified using the definition by the Ministry of Finance and Public Credit and Ministry of Health of Mexico in 2014 [31], which considers two criteria: an energy density of > 1151 kJ/100g (275 kcal/100g) and to be classified as "nonessential foods”. NEDF were classified in four groups according to nutrition composition and consumption patterns: 1) Chips and popcorn, 2) Sweet bakery products, 3) Non-cereal based sweets and, 4) Ready-to-eat cereals. Subgroups of these principal groups were constructed to obtain more homogenous groups as presented in Table 1. We excluded salty seeds or other seed products from the first group because there is solid evidence that seeds are associated with weight lost and are considered a healthy food [32–34]; they were included in the tax because of their high sodium content but our outcome of interest was weight gain and not sodium-related outcomes. Also, we classified beverages into the following food groups: plain water, sugar-sweetened beverages (regular soda, homemade fruit water with added sugar, sweetened milk, coffee or tea with sugar, fruit drinks and sport beverages), 100%-fruit juice, and milk without sugar.

#### Covariates

A *socioeconomic index* was calculated at baseline using a questionnaire administered to the head of household that included sanitation and household characteristics and assets. Using this information, we generated an index using principal components analysis. *Maternal BMI* was calculated using measured weight and height; *maternal formal education* was categorized as less than secondary school, secondary school, and high school or higher. *Marital status* was categorized as single (single, separated, divorced or widower participants) and marriage or free union (participants in marriage or living together with a couple). *Maternal age* was obtain using birth date, while children’s sex was obtained from the birth certificate.

##### Physical activity and sedentary activities.

Physical activity and sedentary activities were assessed at 7 and 11 years old, using a validated semi-quantitative questionary based on the Youth Activity Questionnaire developed and validated by Hernández et al [35]. Physical activity included: playing soccer, volleyball, cycling, skating or skateboarding, basketball, dancing, swimming, walking, taking care of pets, cleaning the house, playing games at home or in the school, among others. Sedentary activities included: time spend watching television, playing videogames, reading, and doing homework and was defined using the time doing this activity during weekdays and weekends. Available responses included: “0 h”, “<0.5 h”, “0.5–2 h”, “2–4 h”, “4–6 h”, “> 6 h” and responses were scored “0 h”, “0.25 h”, “1.25 h”, “3 h”, “5 h”, and “6 h”, respectively. Items from physical activity and sedentary activities were added to obtain the total minutes in a week and then per day.

### Statistical methods

Children’s age and sex and mother’s age, education, marital status, and BMI were described at baseline. We evaluated trends across the three cohort waves using grams, joules (calories), percent total energy of NEDF and its subgroups and for BMI. As physical activity and sedentary behavior was just measure at 7 and 11 years, we assumed no change from 5 to 7 years old, imputing for age 5 the same values of age 7. Then we test for trend for dietetic, anthropometric and physical activity variables across 5, 7 and 11 years using an extension of the Wilcoxon rank-sum test created for this purpose. Fixed effect models were used to assess the association between within-individual change in NEDF consumption and the change in BMI. Fixed effect models considered the nesting structure of data waves nested within children, using age as time of observation [36]. Energy from NEDF and its subgroups was rescaled to produce coefficients relative to 418.6 kJ (100 kcal) change. We ran three different models for each main exposure variable. The first model was unadjusted, the second model was controlled by change in joules different from the main exposure (joules/day) [37] and the third was adjusted by model 2, plus change in physical activity (min/day) and change in tv watching (min/day).

Some sensitivity analyses were performed to assess the robustness of the results. First, in the fixed effect model we changed the adjustment of change in energy different from NEDF for those dietary groups that are closely related with change of BMI, like sugar-sweeten beverages, 100% fruit juice, milk, fruits, dairy food, meat and eggs and processed meet. Second, we left the raw value of the detected dietary outliers to understand their influence in our results. Finally, we changed our model from fixed to a random effects model to estimate the association between BMI and NEDF consumption using the confounders that change in time and without changing in time. All statistical analyses were performed in STATA^®^ Version 13 [38].

## Results

**Fig 1.**
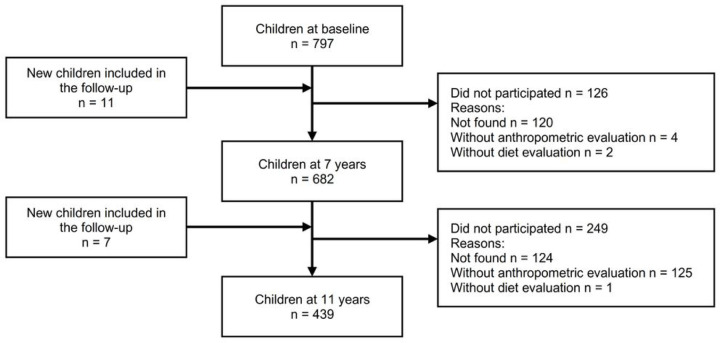
Flowchart of school-aged children at baseline, seven and eleven years old in the cohort study.

The baseline wave was conducted when children were 5 years old (between 2010 and 2011), with the first follow-up at 7 years (between 2012 and 2013) and second at 11 years (between 2015 and 2016). The study sample consisted of 797 at baseline, 682 at 7 years and 439 at 11 years old. Reasons for follow-up losses are presented in the flowchart (Fig. 1). Table 2 presents the characteristics of children and their mothers participating in our study. At baseline there were 797 children and 47% were female. Of them, 85.9% were followed to second (7 years) and 70.7% to the third wave (11 years). Children’s mothers were on average 31.4 years old and majority of them (78.7%) had less than high school, were not single, and overweight.

Children contributed with 1885 data points, which represents and average of 2.4 visits per child over a mean of 6.1 years of follow-up. Table 3 presents the longitudinal change in body weight, BMI, other anthropometric and physical indicators. Mean BMI increased in the first period 1.0 ± 1.46 kg/m^2^ and 4.07 ± 2.9 kg/m^2^ in the second; this represents an increase of 0.56 ± 0.96 in BMI z-score and 24.2% increase in the proportion of overweight and obesity from baseline to the third wave.

Mean total caloric intake at baseline was 6086.4 kJ (1454 kcal), 19.6% of those joules were NEDF. At baseline 95% of children consumed NEDF in the previous day of assessment, 92% by age 7, and 88% by age 11 (data not shown). At baseline, beverage energy intake represented 20% of the total caloric intake; 5.6% of milk, 14.2% of sugar sweetened beverages and a minimal proportion from juice, tea without sugar and other beverages (Supplemental table 1). Table 4 presents baseline and change levels of NEDFL consumption in joules (calories), grams and percentage of total energy consumption to 7 and 11 years old. All sweet bakery products, especially sweet bread, were the main contributors to NEDF with 644 kJ/day (154 kcal/day) (10.5% of total caloric intake). Non-cereal based sweets, ready-to-eat cereals, and chips and popcorn represented a total caloric intake of 3.8%, 3.1% and 2.1%, respectively. On average, the amount of NEDF consumption increased 113 ± 1289 kJ/day (27 ± 308 kcal/day) from baseline to the first wave and 226 ± 1536 kJ (54 ± 367 kcal/day) from baseline to the second wave (p = 0.034); nevertheless, total caloric intake decreased by 2.3 and 3.1 percent points, respectively. In all NEDF groups and subgroups, except for ready-to-eat cereals, the amount in grams and caloric intake increased from 5 to 11 years, yet, as a percent of total energy the change was either negative or null. Consumption of chips and popcorn increased in joules [∆ 98 kj/day (∆ 23.4 kcal/day)] and percent total energy (∆ 0.7 pp/wave) from 5 to 11 years old. Sweet bakery consumption increased on average 54 ± 1050 kJ (13 ± 251 kcal) in first period and 129 ± 1213 kJ (31 ± 290 kcal) in the second, mainly due to sweet bread.

Table 5 includes the unadjusted and adjusted association between BMI change and NEDF change. In the fully adjusted model, the increase in 418.6 kJ/day (100 kcal/day) in total NEDF was associated with a 0.033 kg/m^2^ increase in children’s BMI (95% CI: −0.023, 0.090; p = 0.246). The effect across NEDF subgroups was heterogeneous. The effect between sweet bakery products and BMI was 0.061 kg/m^2^ in the unadjusted model (95%CI: −0.012, 0.135, p = 0.102), increasing to 0.078 kg/m^2^ in the fully adjusted model (95%IC: 0.005, 0.151; p = 0.035). In the stratified subgroups of sweet bakery products, 418.6 kJ/day (100kcal/day) increase of whole grain bread with added sugar (−0.176 kg/m^2^ [95%IC: −0.394, 0.041; p = 0.113]) and cake and pie consumption (0.050 kg/m^2^ [95%IC: −0.061, 0.162; p = 0.375]) were in the expected direction, although not statistically significant. But 418.6 kJ/day (100 kcal/day) increase in sweet bread was associated with a 0.127 kg/m^2^ BMI increase (95%IC: 0.036, 0.218; p = 0.006). Also, in the unadjusted model, 418.6 kJ/day (100kcal/day) increase in chips and popcorn intake was associated with a 0.208 kg/m^2^ BMI increase (95%IC: 0.036, 0.380; p = 0.017), being attenuated in the fully adjusted model (0.086 kg/m^2^ [95%IC: −0.078, 0.252; p = 0.303]). Coefficients for association between consumption of non-cereal based sweets and ready-to-eat cereals were negatively associated with children’s BMI. Non-cereal based sweets consumption was not significantly associated even in the stratified subgroups of cocoa and other sweets. Ready-to-eat cereals in the unadjusted model was inverse associated with BMI (−0.095 [95%CI: −0.305, −0.152]; p = 0.030) but after dietary and physical activity adjustment the coefficient went lower and p value lost its significance (−0.095 [95%CI: −0.233, 0.043] p = 0.178).

In further sensitivity analyses, adjustment for those dietary groups that are closely related with change of BMI (Supplemental table 2) and not truncating outlier truncation, did not change results of the models. In the random effect analysis, we found a similar pattern of association, with weaker coefficients for most of the outcomes. Chips and popcorn, sweet bakery products and sweet bread were associated for each 418.6 kJ/day (100kcal/day) increase in consumption with a 0.128 kg/m^2^ (95%IC: 0.006, 0.250; p = 0.039), 0.055 kg/m^2^ (95%IC: 0.018, 0.108; p = 0.043) and 0.070 kg/m^2^ (95%IC: 0.006, 0.135; p = 0.032) BMI increase, respectively (Supplemental table 3).

## Discussion

We aimed to estimate the association between changes in NEDF consumption and BMI change in school-age children. Over an average of 6.1 years of follow-up children increased their NEDF consumption by 225 kJ (53.9 kcal), yet, we did not observe an association between NEDF increases and BMI increase (0.033 kg/m2, [p = 0.246]). However, in fully adjusted models BMI increased 0.078 kg/m^2^ for every 418.6 kJ/day (100 kcal/day) of sweet bakery products intake (p = 0.035). Increases in the consumption of chips and popcorn were associated with a 0.208 kg/m^2^ increase in BMI in unadjusted models; however, this association was attenuated in fully-adjusted models. Changes in the consumption of other food groups within the NEDF classification did not show an association with changes in BMI.

Few efforts have been made to estimate the association between NEDF consumption and weight in school-aged children. Some cross-sectional studies have described a positive association between NEDF and weight gain [12, 39], however, the few longitudinal studies available have shown mixed results. In two studies, the first under five years of age and the second with a mean age of 16 years, children’s weight tended to decrease with higher levels of consumption of NEDF [19, 20]. A positive association was observed in a study of 961 children 5 to 12 years of age, that defined dietary patterns rich in high-energy and low-nutrient-density foods as exposure [21] with 2.5 years follow-up. Similarly, Phillips, et al, found a non-significative result that children’s weight tend to increase with more joules (calories) from NEDF consumption in a cohort of 166 non-overweight school-aged girls followed for seven years [22], both of these cohort studies used mixed-effect models which can not control for time-invariant confounders as effectively as fixed effects models. Our study is unique in that we are assessing the impact of changes in NEDF consumption and changes in BMI; under this approach we did not detect an association between NEDF and BMI overall, but we did identify a significant association with the consumption of sweet bakery products.

The association between sweet bakery product consumption and weight gain has been reported in two other prospective studies. Phillips *et al.*, in a girls’ cohort study with an average follow-up of seven years, found an increment of z-score in BMI (0.003; p = 0.11), with more intake of cookies, pies, cakes, brownies, chocolate candy, nonchocolate candy, ice cream, milkshakes sherbet, potato chips and corn chips [22]. The non-significant result for potato chips and popcorn un our study could be explained by a lack of power to detect the effect due to a small sample size and the small number of energy-dense foods included in the food frequency questionary. In the preset study we identified more than 90 different types of NEDF in children’s diet. Also, in a prospective cohort study with more than 120 thousand adults followed for 20 years the consumption of one serving of potato chips per day or refined grains (including sweet bakery products) was associated with increment in body weight of 0.77 kg and 0.25 kg in a 4-year period, to each food product, respectively [34]. Negative associations between chips and bakery and BMI have also been reported. Field et al., in a cohort of children and adolescents with three years of follow-up found a reduction of 0.006 in BMI z-score (p < 0.05) among those eating energy-dense foods; however, this association became non-significant after adjusting for dieting status and maternal overweight [23].

In our study we found that eating ready-to-eat cereals was marginally associated with a BMI reduction of −0.098 kg/m^2^ for every 418.6 kJ/day (100 kcal/day). Consumption of ready-to-eat cereals has been related to a healthy dietary pattern in children [40] and this include more consumption of vitamins and minerals, less of saturated fat and cholesterol, but also, with a higher intake of added sugar [40, 41]. Negative associations between ready-to-eat cereals and BMI, have also been reported in longitudinal analyses [42, 43]. However, ready-to-eat cereals comprises many different products, and their nutritional impact will depend on the composition of the cereal, and the food consumed with them (such as fruit or milk); our study, as well as all previous studies available could be confounded by these characteristics. Even though ready-to-eat cereals may be associated with weight lost in children, children consuming a non-high fiber cereal, had worse type 2 diabetes risk profile than children consuming a high fiber cereal in a longitudinal study [44].

There are three different explanations for the association between sweet bakery products consumption and weight gain. First, sweet bakery products tend to be high in added sugar, saturated fat and of course high quantities of energy in small portions of food [10] and may promote excess energy intake without control over the joules (calories) consumed. In a recent crossover trial, adults were randomized to receive an ultra-processed (generally energy-dense food) or unprocessed diet for a period of 2 weeks. Participants in the ultra-processed diet increased 0.8 kg and those in the unprocessed diet reduced 1.1 kg. Those in the ultra-processed diet consumed 2126 kJ/day (508 kcal/day) more than the other group, mainly by fats and carbohydrates [45]. Second, many sweet bakery products are high in refined carbohydrates and starches and may induce stronger insulin secretion. This promotes less satiating signals, increasing subsequent hunger feelings [46] and suppresses the release of fatty acids from adipose tissue into circulation, while keeping glucose and fatty acids away from the oxidation process to store them in the adipose tissue [47]. Third, a diet rich in energy-dense food is associated with less protein consumption in children and according with the “protein leverage hypothesis” this may be related to the disturbance of the appetite system through increased postprandial hunger and reduced postprandial satiety [48].

Strengths of this study include the prospective cohort design, including three measurements of anthropometric and dietary information, large sample size and the approach analysis with the possibility to assess the change in change effect. Our study also has some important limitations that must be taken into account to interpret our results. First, at each wave we had one dietary evaluation, instead of two or more 24-hour recalls, which may not reflect usual NEDF consumption. Second, we lost 14.1% of the sample in the first period and 29.3% in the second; however, baseline socioeconomic index, children sex at baseline, age and maternal education, and overweight status were no different between children lost and those who stayed in the cohort.

In summary, our results showed that consumption of NEDF overall was not associated with BMI in children. However, NEDF subgroups such as sweet bakery products and, possibly, chips and popcorn, showed an association with BMI. The longer-term effect of NEDF consumption in school-aged children on BMI requires further studies with bigger samples, better follow-up, and the use of an objective measure of adipose tissue in order to obtain more reliable results. Decreasing the consumption of NEDF is a key step to improve dietary quality and prevent obesity, aligned with the WHO 25x25 goals [49] and the Sustainable Development Goals [50]. In Mexico, a strategy to reduce NEDF consumption is in place, limiting access to these foods in elementary schools [51], restricting food marketing to children on television and public areas [52], and implementing an 8% tax to all NEDF [31]. However, further public health efforts need to be directed to reduce the consumption of NEDF, particularly early on in life.

## Supplementary Material

Supplement 1

## Figures and Tables

**Table 1:** Classification of nonessential energy dense food.

Main groups of NEDF^1^	Subgroups of NEDF^1^	Food examples
Chips and popcorn		Fried potato, flour and corn chips, packaged fried pork skin, ready-to-eat popcorn, microwave popcorn.
Sweet bakery products	Whole grain with added sugar	Bars, enriched bread and cookies made with whole grain flour but all with added sugar.
	Sweet bread	Sweet cookies, sweet bread, energy bars and cereal bars.
	Pie and cakes	All kinds of pies and cakes.
Non-cereal based sweets	Cocoa and other sweet products	Cocoa, raisin, plum and legumes covered with chocolate and gums with or without sugar.
	Sweets	Strawberry, vanilla or chocolate powder, condensed milk, fruit preserves, candies, marshmallows, jam, jellies, “dulce de leche” or “cajeta”, hazelnut spread, caramels, ice cream, ice-pops, popsicle.
Ready-to-eat cereals		All the pre-prepared and ready-to-eat cereal with added sugar.

1 NEDF, Nonessential energy-dense food.

**Table 2:** Descriptive characteristics of children and their mothers in the POSGRAD^1^ study at baseline.

Demographic characteristics	Baseline (n = 797)
**Children age (mean ± SD)**	
Years	4.91 ± 0.28
**Children sex (n/%)**	
*Male*	424 (53)
*Female*	373 (47)
**Mother's age (mean ± SD)**	31.3 ± 4.8
**Mother's education (n/%)**	
*Less than secondary school*	303 (38.1)
*Secondary and high school*	323 (40.6)
*More than high school*	170 (21.3)
**Mother's civil status (n/%)**	
Single	72 (9.0)
Married or free union	725 (91.0)
**Mother's BMI (mean ± SD)**	26.15 ± 4.3
Normal weight (n/%)	342 (42.9)
Overweight or obese (n/%)	455 (57.9)

1 **POSGRAD**, Prenatal omega-3 fatty acid supplementation and child growth and development.

SD, standar deviation.

**Table 3:** Mean change in anthropometric measurements, nutritional status and physical activity in children from baseline to 7 and 11 years old.

	∆ from baseline to		
	Baseline (n = 797)	7 years (n = 682)	11 years (n = 439)
**Weight (kg)**	18.3 ± 2.9^2^	6.30 ± 2.95	23.46 ± 8.4
**Height (cm)**	108.3 ± 4.4^2^	13.17 ± 1.9	37.09 ± 4.2
**Body mass index (kg/m** ^**2**^ **)**	15.5 ± 1.7^2^	1.00 ± 1.46	4.07 ± 2.9
**Height for age, z score**	−0.39 ± 0.9^2^	0.25 ± 0.24	0.48 ± 0.54
**Body mass index for age,** z**score**	0.11 ± 1.1^2^	0.30 ± 0.69	0.56 ± 0.96
Underweight (n/%)	9 (1.1)	10 (1.5)	16 (3.7)
Normal weight (n/%)	639 (80.2)	465 (68.2)	335 (53.5)
Overweight (n/%)	101 (12.7)	114 (16.7)	107 (24.4)
Obesity (n/%)	48 (6.0)	93 (13.6)	81 (18.5)
**Physical activity (min/day)** ^1^	ND	74.9 ± 46.3^2^	−12.6 ± 57.1
**Total sedentary activities (min/day)** ^1^	ND	252.4 ± 104.8^2^	39.25 ± 143.9
TV	ND	115.5 ± 65.4^2^	−16.5 ± 82.2
Other sedentary activities	ND	137.02 ± 68.6^2^	55.8 ± 110.2

Results are presented in means change and standard deviation, unless it specifies different.

1 n=677 at 7 years and n = 399 at 11 years old.

ND, No data.

Test for trend across 5, 7 and 11 years old.

2 p value < 0.001.

**Table 4:** Mean change in grams, joules (calories) and percentage of total calorie intake of nonessential and energy-dense food from baseline to 7 and 11 years old.

	∆ from baseline to								
	Baseline (n = 797)			7 years (n = 682)			11 years (n = 439)		
	g	kJ (kcal)	ptec^1^	∆ g	∆ kJ (kcal)	∆ ptec^1^	∆ g	∆ kJ (kcal)	∆ ptec^1^
**Total NEDF**	73 ± 58^2^	1188 ± 925^2^ (284 ± 221)	19.6 ± 13^3^	6.8 ± 82	111 ± 1289 (26.6 ± 308)	−2.3 ± 17	13.7 ± 94	225 ± 1536 (53.9 ± 367)	−3.1 ± 18
**Chips and ready-to-eat popcorn**	6 ± 14^3^	133 ± 293 ^3^ (32 ± 70)	2.1 ± 4.8 ^3^	1.1 ± 22	24 ± 468 (5.9 ± 112)	−0.1 ± 6.8	5.1 ± 25	97 ± 514 (23.4 ± 123)	0.7 ± 7.3
**Sweet bakery products**	41 ± 49 (154 ± 175)	644 ± 732	10.5 ± 12^2^	3.2 ± 72 (12.8 ± 252)	53 ± 1054	−1.3 ± 15	9.3 ± 85	128 ± 1218 (30.8 ± 291)	−1.7 ± 15
Whole-cereal with sugar	5 ± 14 (21 ± 59)	87 ± 246	1.4 ± 4.0	0.8 ± 20 (3.1 ± 82.7)	12 ± 346	−0.1 ± 5.4	1.4 ± 29 (5.4 ± 118)	22 ± 493	−0.21 ± 5.5
Sweet bread	25 ± 37	418.6 ± 623 (100 ± 149)	6.8 ± 9.8	6.9 ± 55	95 ± 895 (22.7 ± 214)	0.0 ± 13	6.1 ± 53	84 ± 866 (20.2 ± 207)	−0.7 ± 12
Cake, pie and others	11 ± 35	138 ±422 (33 ± 101)	2.3 ± 7.4	−4.3 ± 52	−52 ± 632 (−12.6 ± 151)	−1.3 ± 9.5	1.9 ± 66	23 ± 799 (5.5 ± 191)	−0.8 ± 9.4
**Non-cereal based sweets**	14 ± 20^2^	226 ± 309 ^2^ (54 ± 74)	3.8 ± 4.8 ^3^	0.7 ± 30	5 ± 481 (1.3 ± 115)	−0.6 ± 6.7	2.3 ± 35	35 ± 539 (8.6 ± 129)	−0.8 ± 7.0
Cocoa and legumes with sugar	0.3 ± 2^3^	3 ± 20 ^3^ (0.9 ± 5)	0.1 ± 0.4 ^3^	−0.1 ± 3	−1 ± 46 (−0.3 ± 11)	−0.0 ± 0.7	0.3 ± 8.9	3 ± 108 (0.9 ± 26)	0 ± 1.4
Sweets	14 ± 20^2^	226 ± 305 ^2^ (54 ± 73)	3.7 ± 4.8 ^3^	0.7 ± 30	5 ± 481 (1.3 ± 115)	−0.6 ± 6.7	1.9 ± 34	30 ± 531 (7.3 ± 127)	−0.8 ± 6.9
Ready-to-eat cereals	12 ± 24^3^	179 ± 376 ^3^ (43 ± 90)	3.1 ± 6.3 ^3^	1.7 ± 38	27 ± 602 (6.6 ± 144)	−0.2 ± 8.4	−2.4 ± 35	−36 ± 544 (−8.8 ± 130)	−1.2 ± 7.2

Results are presented in means change and standard deviation.

Test for trend across 5, 7 and 11 years old.

1 ptec: percentage of total energy consumption.

2 p value < 0.05

3 p value < 0.001

**Table 5:** Mean change in body mass index associated with total change consumption of NEDF and its subgroups in school age children from 5 to 11 years old.

Main exposure	Model	Coefficient	95% CI	*p*
**Total NEDF**	1	0.039	−0.018, 0.098	0.184
	2	0.030	−0.025, 0.086	0.282
	3	0.033	−0.023, 0.090	0.246
**Chips and popcorn**	1	0.208	0.036, 0.380	0.017
	2	0.160	−0.005, 0.325	0.058
	3	0.086	−0.078, 0.252	0.303
**Sweet bakery products**	1	0.061	−0.012, 0.135	0.102
	2	0.096	−0.010, 0.130	0.096
	3	0.078	0.005, 0.151	0.035
Whole-grain with added sugar	1	−0.056	−0.252, 0.139	0.569
	2	−0.175	−0.365, 0.014	0.070
	3	−0.176	−0.394, 0.041	0.113
Sweet bread	1	0.112	−0.017, 0.207	0.020
	2	0.123	0.032, 0.214	0.008
	3	0.127	0.036, 0.218	0.006
Pie and cakes	1	0.006	−0.109, 0.122	0.911
	2	0.024	−0.086, 0.135	0.667
	3	0.050	−0.061, 0.162	0.375
**Non-cereal based sweets**	1	0.031	−0.129, 0.192	0.701
	2	0.839	−0.170, 0.138	0.839
	3	−0.029	−0.181, 0.123	0.705
Cocoa and other sweet products	1	−0.054	−1.296, 1.187	0.931
	2	−0.122	−1.314. 1.069	0.840
	3	−0.131	−1.284,1.022	0.824
Sweets	1	0.028	−0.133, 0.1912	0.729
	2	−0.016	−0.172, 0.139	0.834
	3	−0.029	−0.183, 0.123	0.702
**Ready-to-eat cereals**	1	−0.160	−0.305, −0.152	0.030
	2	−0.107	−0.247, 0.032	0.131
	3	−0.095	−0.233, 0.043	0.178

NEDF, nonessential and energy-dense food. Coefficients are presented in units of change of 418.6 kJ/(100 kcal/day).

Fixed effects models: Model 1 (n = 724), unadjusted; Model 2 (n = 724): adjusted by change in joules different from the main exposure [kJ/day (kcal/day)); Model 3 (n = 602): adjusted by model 2 + change in physical activity (min/day) and change in tv watching (min/day).

## Abbreviations

BMI: body mass index
NEDF: nonessential energy-dense food
POSGRAD: Prenatal omega-3 fatty acid supplementation, child growth, and development
24HR: 24-hour recall

## References

[1] Ng M, Fleming T, Robinson M, Thomson B, Graetz N, Margono C, Global, regional, and national prevalence of overweight and obesity in children and adults during 1980–2013: a systematic analysis for the Global Burden of Disease Study 2013. Lancet. 2014;384:766–81. 24880830 10.1016/S0140-6736(14)60460-8 PMC4624264

[2] Bentham J, Di Cesare M, Bilano V, Bixby H, Zhou B, Stevens GA, Worldwide trends in body-mass index, underweight, overweight, and measurement studies in 128.9 million children, adolescents, and adults. Lancet. 2017;319:2627–42. 10.1016/S0140-6736(17)32129-3 PMC5735219 29029897

[3] Hernández-Cordero S, Cuevas-Nasu L, Morales-Ruán M, Humarán IM-G, Ávila-Arcos M, Rivera-Dommarco J. Overweight and obesity in Mexican children and adolescents during the last 25 years. Nutr Diabetes. 2017;7:e247. 28287630 10.1038/nutd.2016.52 PMC5380891

[4] Shamah-Levy T, Romero-Martínez M, Barrientos-Gutiérrez T, Cuevas-Nasu L, Bautista-Arredondo S, Colchero M, Encuesta Nacional de Salud y Nutrición 2021 sobre Covid-19. Resultados nacionales. Cuernavaca, México: Instituto Nacional de Salud Pública; 2022.

[5] Franks PW, Hanson RL, Knowler WC, Sievers ML, Bennett PH, Looker HC. Childhood obesity, other cardiovascular risk factors, and premature death. New Eng J Med. 2010;362:485–93. 20147714 10.1056/NEJMoa0904130 PMC2958822

[6] Dwyer J. Starting down the right path: nutrition connections with chronic diseases of later life. Am J Clin Nutr. 2006;83:415S–20S. 16470005 10.1093/ajcn/83.2.415S

[7] Halldorsson TI, Gunnarsdottir I, Birgisdottir BE, Gudnason V, Aspelund T, Thorsdottir I. Childhood Growth and Adult Hypertension in a Population of High Birth Weight. Hypertension. 2011;58:8–15. 21576624 10.1161/HYPERTENSIONAHA.111.170985

[8] Reilly JJ, Kelly J. Long-term impact of overweight and obesity in childhood and adolescence on morbidity and premature mortality in adulthood: systematic review. Int J Obes (Lond). 2011;35:891–8. 20975725 10.1038/ijo.2010.222

[9] Sánchez-Pimienta TG, Batis C, Lutter CK, Rivera JA. Sugar-sweetened beverages are the main sources of added sugar intake in the Mexican population. J Nutr. 2016;146:1888S–96S. 27511931 10.3945/jn.115.220301

[10] Batis C, Pedraza LS, Sánchez-Pimienta TG, Aburto TC, Rivera-Dommarco JA. Energy, added sugar, and saturated fat contributions of taxed beverages and foods in Mexico. Salud Publ Méx. 2017;59:512–7. 10.21149/8517 29267647

[11] Romieu I, Dossus L, Barquera S, Blottière HM, Franks PW, Gunter M, Energy balance and obesity: what are the main drivers? Cancer Causes Control. 2017;28:247–58. 28210884 10.1007/s10552-017-0869-z PMC5325830

[12] Larson NI, Miller JM, Watts AW, Story MT, Neumark-Sztainer DR. Adolescent snacking behaviors are associated with dietary intake and weight status. J Nutr. 2016;146:1348–55. 27281807 10.3945/jn.116.230334 PMC4926852

[13] Cornwell B, Villamor E, Mora-Plazas M, Marin C, Monteiro CA, Baylin A. Processed and ultra-processed foods are associated with lower-quality nutrient profiles in children from Colombia. Public Health Nutr. 2018;21:142–7. 28554335 10.1017/S1368980017000891 PMC10260756

[14] Aburto TC, Pedraza LS, Sánchez-Pimienta TG, Batis C, Rivera JA. Discretionary foods have a high contribution and fruit, vegetables, and legumes have a low contribution to the total energy intake of the Mexican population. J Nutr. 2016;146:1881S–7S. 27511928 10.3945/jn.115.219121

[15] Bonvecchio-Arenas A, Theodore FL, Hernández-Cordero S, Campirano-Núñez F, Islas AL, Safdie M, The school as an opportunity for obesity prevention: An experience from the Mexican school system. Rev Espanola de Nutr Comunitaria. 2010;16:13–6.

[16] Ramírez-Ley K, De Lira-García C, de las Cruces Souto-Gallardo M, Tejeda-López MF, Castañeda-González LM, Bacardí-Gascón M, Food-related advertising geared toward Mexican children. J Public Health. 2009;31:383–8. 10.1093/pubmed/fdp058 19531604

[17] Calvert SL. Children as consumers: Advertising and marketing. Future Child. 2008;18:205–34. 21338011 10.1353/foc.0.0001

[18] Prentice AM, Jebb SA. Fast foods, energy density and obesity: a possible mechanistic link. Obes Rev. 2003;4:187–94. 14649369 10.1046/j.1467-789x.2003.00117.x

[19] Cunha DB, Costa THM, Veiga GV, Pereira RA, Sichieri R. Ultra-processed food consumption and adiposity trajectories in a Brazilian cohort of adolescents: ELANA study. Nutr Diabetes. 2018;8:28. 29795367 10.1038/s41387-018-0043-z PMC5968026

[20] Durão C, Severo M, Oliveira A, Moreira P, Guerra A, Barros H, Evaluating the effect of energy-dense foods consumption on preschool children’s body mass index: a prospective analysis from 2 to 4 years of age.. Eur J Nutr. 2015;54:835–43. 25185968 10.1007/s00394-014-0762-4

[21] Shroff MR, Perng W, Baylin A, Mora-Plazas M, Marin C, Villamor E. Adherence to a snacking dietary pattern and soda intake are related to the development of adiposity: a prospective study in school-age children. Public Health Nutr. 2014;17:1507–13. 23701749 10.1017/S136898001300133X PMC10282458

[22] Phillips SM, Bandini LG, Naumova EN, Cyr H, Colclough S, Dietz WH, Energy‐dense snack food intake in adolescence: longitudinal relationship to weight and fatness.. Obes Res. 2004;12:461–72. 15044663 10.1038/oby.2004.52

[23] Field AE, Austin SB, Gillman MW, Rosner B, Rockett HR, Colditz GA. Snack food intake does not predict weight change among children and adolescents. Int J Obes. 2004;28:1210–16. 10.1038/sj.ijo.0802762 15314623

[24] Gonzalez-Casanova I, Stein AD, Hao W, Garcia-Feregrino R, Barraza-Villarreal A, Romieu I, Prenatal Supplementation with Docosahexaenoic Acid Has No Effect on Growth through 60 Months of Age–3. J Nutr. 2015;145:1330–4. 25926416 10.3945/jn.114.203570 PMC4442112

[25] Lohman T, Roche A, Martorell R. Anthropometric Standardization Reference Manual Abridged Edition: Human Kinetics Books; 1991. Available from: http://books.google.com.mx/books?id=wgd9QgAACAAJ.

[26] World Health Organization. WHO AnthroPlus for personal computers manual: software for assessing growth of the world’s children and adolescents. Geneva: WHO. 2009). Available from: https://www.who.int/growthref/tools/en/.

[27] De Onis M. WHO child growth standards: Methods and development - Length/Height-for-age, Weight-for-age, Weight-for-length, Weight-for-height and Body mass index-for-age 2006). Available from: https://www.who.int/childgrowth/standards/Technical_report.pdf?ua=1.

[28] Angulo-Estrada JS, Espinosa-Montero J, Gaytan-Colin MA, González-de-Cossío-Martínez T, Gutiérrez JP, Barrera LH, Programa de Cómputo: Rec24Hrs. 5 Pasos (R24H5). Cuernavaca, Morelos: Instituto Nacional de Salud Pública; 2013.

[29] Conway JM, Ingwersen LA, Vinyard BT, Moshfegh AJ. Effectiveness of the US Department of Agriculture 5-step multiple-pass method in assessing food intake in obese and nonobese women. Am J Clin Nutr. 2003;77:1171–8. 12716668 10.1093/ajcn/77.5.1171

[30] Ramírez Silva I, Barragán-Vázquez S, Rodríguez-Ramírez S, Rivera-Dommarco J, Mejía-Rodríguez F, Barquera-Cervera S, Base de alimentos de México (BAM): Compilación de la composición de los alimentos frecuentemente consumidos en el país. 2019.

[31] Congreso de los Estados Unidos Mexicanos. Ley del Impuesto Especial sobre Producción y Servicios Mexico2014 [Available from: http://www.diputados.gob.mx/LeyesBiblio/pdf/78_241219.pdf.

[32] Bitok E, Sabate J. Nuts and Cardiovascular Disease. Prog Cardiovasc Dis. 2018;61:33–7. 29800597 10.1016/j.pcad.2018.05.003

[33] Kris-Etherton PM, Hu FB, Ros E, Sabaté J. The role of tree nuts and peanuts in the prevention of coronary heart disease: multiple potential mechanisms. J Nutr. 2008;138:1746S–51S. 18716180 10.1093/jn/138.9.1746S

[34] Mozaffarian D, Hao T, Rimm EB, Willett WC, Hu FB. Changes in diet and lifestyle and long-term weight gain in women and men.. New Eng J Med. 2011;364:2392–404. 21696306 10.1056/NEJMoa1014296 PMC3151731

[35] Hernández B, Gortmaker SL, Laird NM, Colditz GA, Parra-Cabrera S, Peterson KE. Validez y reproducibilidad de un cuestionario de actividad e inactividad física para escolares de la ciudad de México. Salud Publica Mex. 2000;42:315–23. 11026073

[36] Singer JD, Willett JB. Applied longitudinal data analysis: Modeling change and event occurrence: Oxford university press; 2003.

[37] Hu FB, Stampfer MJ, Rimm E, Ascherio A, Rosner BA, Spiegelman D, Dietary fat and coronary heart disease: a comparison of approaches for adjusting for total energy intake and modeling repeated dietary measurements. Am J Epidemiol. 1999;149:531–40. 10084242 10.1093/oxfordjournals.aje.a009849

[38] StataCorp L. Stata 13: College Station: StataCorp LP; 2014 [Available from: https://www.stata.com/company/.

[39] Juul F, Martinez-Steele E, Parekh N, Monteiro CA, Chang VW. Ultra-processed food consumption and excess weight among US adults. Br J Nutr. 2018;120:90–100. 29729673 10.1017/S0007114518001046

[40] Priebe MG, McMonagle JR. Effects of ready-to-eat-cereals on key nutritional and health outcomes: A systematic review. PLoS One. 2016;11:e0164931. 27749919 10.1371/journal.pone.0164931 PMC5066953

[41] Michels N, De Henauw S, Beghin L, Cuenca-García M, Gonzalez-Gross M, Hallstrom L, Ready-to-eat cereals improve nutrient, milk and fruit intake at breakfast in European adolescents. Eur J Nutr. 2016;55:771–9. 25893716 10.1007/s00394-015-0898-x PMC4767844

[42] Frantzen LB, Treviño RP, Echon RM, Garcia-Dominic O, DiMarco N. Association between frequency of ready-to-eat cereal consumption, nutrient intakes, and body mass index in fourth-to sixth-grade low-income minority children. J Acad Nutr Diet. 2013;113:511–9. 23465566 10.1016/j.jand.2013.01.006

[43] Kuriyan R, Lokesh DP, D'souza N, Priscilla DJ, Peris CH, Selvam S, Portion controlled ready-to-eat meal replacement is associated with short term weight loss: a randomised controlled trial. Asia Pac J Clin Nutr. 2017;26:1055–65. 28917231 10.6133/apjcn.022017.07

[44] Donin AS, Nightingale CM, Owen CG, Rudnicka AR, Perkin MR, Jebb SA, Regular breakfast consumption and type 2 diabetes risk markers in 9-to 10-year-old children in the child heart and health study in England (CHASE): a cross-sectional analysis. PLoS medicine. 2014;11:e1001703. 25181492 10.1371/journal.pmed.1001703 PMC4151989

[45] Hall KD. Ultra-processed diets cause excess calorie intake and weight gain: A one-month inpatient randomized controlled trial of ad libitum food intake. Cell Metab. 2019;30:67–77.e3. 31105044 10.1016/j.cmet.2019.05.008 PMC7946062

[46] Bornet FR, Jardy-Gennetier A-E, Jacquet N, Stowell J. Glycaemic response to foods: impact on satiety and long-term weight regulation. Appetite. 2007;49:535–53. 17610996 10.1016/j.appet.2007.04.006

[47] Hall KD. A review of the carbohydrate–insulin model of obesity. Eur J Clin Nutr. 2017;71:323–6. 28074888 10.1038/ejcn.2016.260

[48] Simpson S, Raubenheimer D. Obesity: the protein leverage hypothesis. Obes Rev. 2005;6:133–42. 15836464 10.1111/j.1467-789X.2005.00178.x

[49] World Health Organization. Global action plan for the prevention and control of noncommunicable diseases 2013–2020. Geneva: World Health Organization. 2015.

[50] Buse K, Hawkes S. Health in the sustainable development goals: ready for a paradigm shift? Global Health. 2015;11:13. 25890267 10.1186/s12992-015-0098-8 PMC4389312

[51] Secretaría de Salud. Acuerdo Nacional para la Salud Alimentaria. Estrategia contra el sobrepeso y la obesidad. México: Secretaría de Salud; 2010.

[52] Consejo de autoregulación y ética publicitariar. Código PABI. Código de autoregulación de publicidad de alimentos y bebidas no alcohólicas dirigida al público infantil. Ciudad de México: CONAR; 2012.

