# Supplementary material for: Association between consumption of nonessential energy-dense food and body mass index among Mexican school-aged children: A prospective cohort study
Source: Res Sq. 2023 Apr 26:rs.3.rs-2833950. Preprint. [Version 1] doi: 10.21203/rs.3.rs-2833950/v1 (PMC10168455; doi:10.21203/rs.3.rs-2833950/v1)
Supplement: Supplement 1 [file NIHPPRS2833950V1-supplement-1.pdf]

Supplementary Files

This is a list of supplementary files associated with this preprint. Click to download.

- Supplementarytables17042023.docx
